# Technical Feasibility and Safety of Repeated Computed Tomography–Guided Transthoracic Intratumoral Injection of Gene-Modified Cellular Immunotherapy in Metastatic NSCLC

**DOI:** 10.1016/j.jtocrr.2021.100242

**Published:** 2021-10-14

**Authors:** Puja Shahrouki, Jay M. Lee, Jonathan Barclay, Sarah N. Khan, Scott Genshaft, Fereidoun Abtin, Steven M. Dubinett, Aaron Lisberg, Sherven Sharma, Edward B. Garon, Robert Suh

**Affiliations:** a Department of Radiological Sciences, David Geffen School of Medicine at University of California, Los Angeles, Los Angeles, California; b Division of Thoracic Surgery, Department of Surgery, David Geffen School of Medicine at University of California, Los Angeles, Los Angeles, California; c Division of Pulmonary and Critical Care Medicine, Department of Medicine, David Geffen School of Medicine at University of California, Los Angeles, Los Angeles, California; d Division of Hematology and Oncology, Department of Medicine, David Geffen School of Medicine at University of California, Los Angeles, Los Angeles, California

**Keywords:** Dendritic cells, Non–small cell lung cancer, Gene therapy, Immunotherapy

## Abstract

**Introduction:**

To assess the technical feasibility and safety of repeated percutaneous computed tomography (CT)–guided transthoracic biopsies and intratumoral injections of gene-modified dendritic cells in metastatic NSCLC.

**Methods:**

A total of 15 patients with 15 NSCLC lesions measuring greater than 1.0 cm underwent two cycles of intratumoral biopsies and CCL21 dendritic cell injections separated by 7 days. All needle placements and injections were done under CT guidance. Clinical and imaging follow-up was done approximately 4 weeks after the first procedure. Safety and feasibility were determined as: (1) safety and feasibility similar to that of single-needle biopsy, and (2) an absence of serious adverse events defined as grade greater than or equal to three according to the National Cancer Institute Common Terminology Criteria for Adverse Events version 5.0.

**Results:**

A total of 30 percutaneous, transthoracic intratumoral biopsies and injections into the lung cancer were performed, two cycles (at d 0 and 7) received by each patient (311 biopsies and 96 intratumoral injections). All percutaneous cases achieved technical success with respect to needle placement for both biopsy and injection of CCL21 dendritic cells. Only minor complications were observed (grade <3), including pneumothorax (n = 10, 33%) and small postbiopsy hemorrhage (n = 2, 7%). Pneumothorax was moderate (n = 1) or trace (n = 9), with resolution of the moderate pneumothorax after manual aspiration without chest tube placement. No patient required chest tube placement. No other complications or serious adverse effects related to the biopsy or dendritic cell injection were noted. All patients were in stable condition after up to 4 hours in the recovery unit and were discharged home on the same day. No procedure-related complications were observed on imaging or clinical follow-up at 4 weeks.

**Conclusions:**

Repeated percutaneous, transthoracic CT-guided biopsies and intratumoral gene-modified cell-based immunotherapy injections into lung cancers are technically feasible, safe, and reproducible. There were no procedure-related serious (defined as grade ≥3) adverse events.

## Introduction

Lung cancer holds a position as the deadliest malignancy for both men and women. NSCLC accounts for most lung cancer cases and carries with it a poor—although improving—2-year survival of 42%.^1^ Treatment for advanced and metastatic NSCLC is of particular interest as most newly diagnosed patients with lung cancer are stage III or IV.^2^

Over the past two decades, immunotherapy has emerged as a promising new tool in the treatment of NSCLC.^3^^,^^4^ Previous studies have revealed that a strong antitumoral response is associated with improved survival in patients with cancer.^5^^,^^6^ Furthermore, the increased presence of CD4^+^ T-cells, CD8^+^ T-cells, natural killer cells, and mature dendritic cells has been associated with better patient survival, specifically in the setting of human NSCLC.^7^^,^^8^ However, tumor cells, such as those in NSCLC, suppress the activity of antigen-presenting cells, leading to poor production of costimulatory signals, ultimately resulting in anergy or peripheral tolerance.^4^^,^^7^^,^^9^ Thus, specific immunotherapies designed to augment the immune response and increase T-cell activation have strong potential as treatment strategies for patients with NSCLC.

One such promising approach uses dendritic cells injected directly at the tumor site.^9^^,^^10^ Dendritic cells, the most potent antigen-presenting cells, have the capacity to modulate systemic immunity.^7^ Through in situ vaccination, the dendric cells have the potential to exploit the tumor-associated antigens at the tumor site to induce a systemic response—essentially creating a vaccine in vivo without having to first isolate the tumor-associated antigen.^11^ An increasing number of preclinical and clinical studies are evaluating the role of intratumoral immunotherapy that is aimed at inducing systemic antitumor responses.^12^^,^^13^

Given the increasingly promising role of immunotherapy in the future of NSCLC treatment, it is important to consider the practicality of strategies such as in situ vaccination. To our knowledge, we were the first to describe the antitumor properties of intratumoral recombinant CCL21 in murine models of NSCLC.^14^ In subsequent preclinical studies, we found that intratumoral administration of dendritic cells overexpressing CCL21 generates systemic antitumor responses and confers tumor immunity.^15^ When we overexpressed CCL-21 in human dendritic cells, we found augmented chemotactic activities for lymphocytes and antigen-presenting cells.^16^

On the basis of these preclinical findings, we conducted the first-in-man evaluation of Ad-CCL21-DC in situ vaccination in advanced NSCLC. This was a phase 1 study of intratumoral administration of autologous dendritic cells transduced with an adenoviral vector expressing the *CCL21* gene (Ad-CCL21-DC vaccine).^17^ Intratumoral Ad-CCL21-DC vaccination resulted in induction of systemic tumor-specific immune responses, enhanced tumor CD8+ T-cell infiltration, and increased tumor programmed death-ligand 1 expression.^17^ Here, we report on the technical feasibility and safety of repeated percutaneous computed tomography (CT)–guided biopsy and intratumoral injections of Ad-CCL21-DC vaccine in patients with metastatic NSCLC.

## Materials and Methods

### Study Population

This study was approved by the institutional review board for a retrospective review of patients with histologically confirmed stage IIIB or IV NSCLC (TNM, seventh edition) who were enrolled in the Ad-CCL21-DC clinical trial (Clinicaltrials.gov: NCT01574222).^17^ Inclusion and exclusion criteria are presented in Supplementary Table 1. The primary target lesions included were greater than 1.0 cm in size, in accessible thoracic locations (e.g., no bullae in the needle path), and amenable to repeated targeted interventions, as determined by the performing interventional radiologist. Written informed consent was obtained from all patients.

A total of 17 patients were enrolled in the study. One patient who underwent bronchoscopic Ad-CCL21-DC injection and one patient who withdrew from the trial were excluded from subsequent analysis, which included a total of 15 patients who underwent two cycles of intratumoral biopsies and Ad-CCL21-DC injections.

### Protocol

Before the scheduled injections, patients received a preprocedural chest CT or had appropriate outside images available for review in the picture archiving and communication system (n = 14). One patient had no previous imaging available for review; however, this patient had the full radiological staging scan reports available for review before the injections.

**Figure 1:**
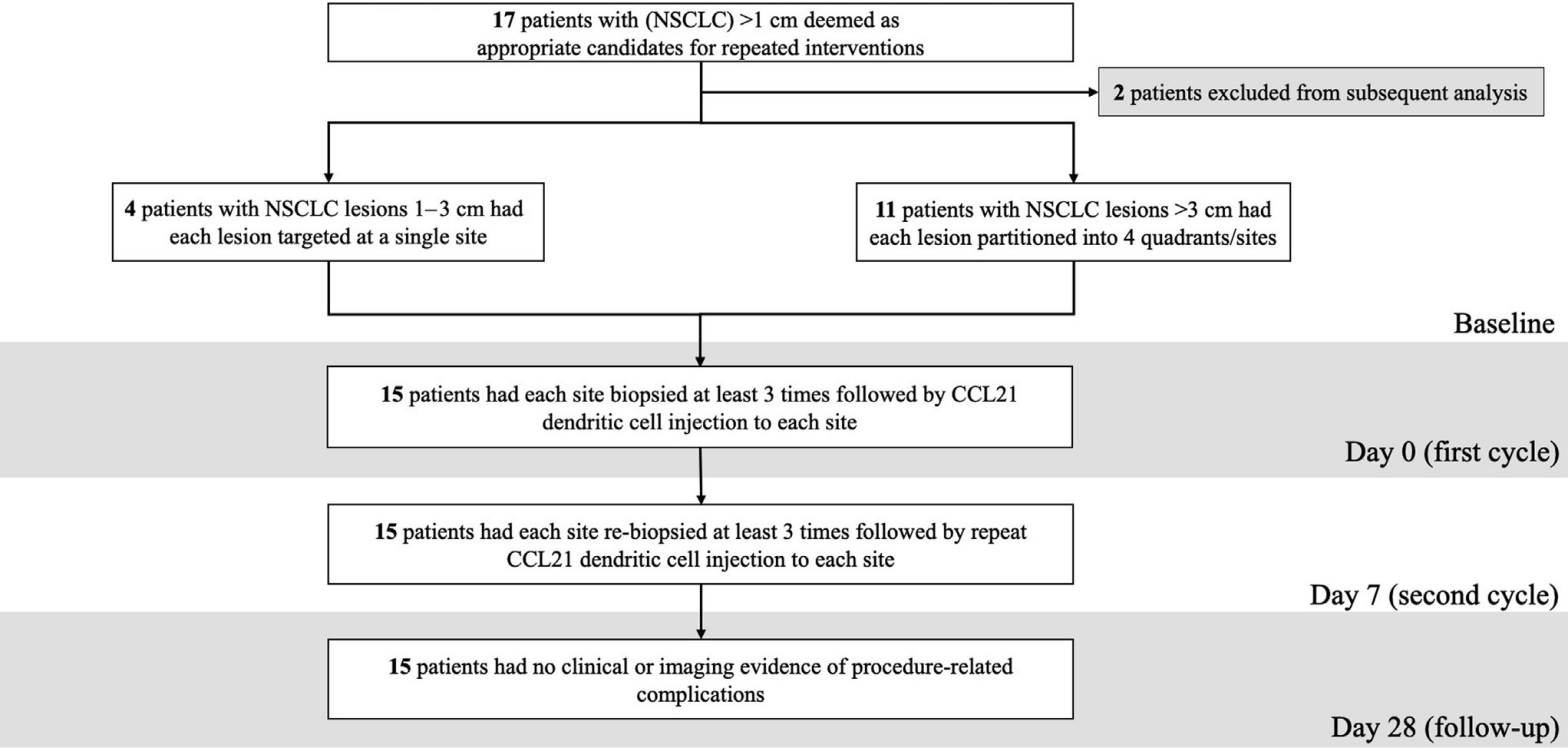
Flowchart of the included study population. Two patients were excluded from the primary safety population: one patient underwent bronchoscopic (not transthoracic) Ad-CCL21-DC injection and one patient withdrew from the trial after the first cycle. The full inclusion and exclusion criteria can be found in Supplementary Table 1.

The preparation of the intratumoral Ad-CCL21-DC injections has previously been described.^17^^,^^18^ All included patients received two intratumoral injections separated by a 7-day interval by one of two experienced interventional radiologists (Fig. 1).^17^

The frequency, dose, and technique were predetermined on the basis of a phase 1 clinical trial (Clinicaltrials.gov: NCT01574222).^17^ Follow-up CT examinations were performed 28 days (± 7 d) after the first intratumoral injection to assess for procedure-related complications and tumor response, as measured and determined by a senior thoracic radiologist. Tumor response was assessed on the basis of the revised Response Evaluation Criteria in Solid Tumors 1.1 guideline.^19^

### Procedure

A limited CT through the target lesion was performed with the patient in the supine (n = 11), supine oblique (n = 2), or prone (n = 2) position to allow for optimal access to the predetermined target lesion. Images were acquired before, during, and after needle placement, including each biopsy and injection site.

Patients were prepared and draped in the usual sterile fashion. The local subcutaneous and soft tissues, and pleural surface were anesthetized with 1% lidocaine (Xylocaine, AstraZeneca International, Wilmington, DE). Each patient’s blood pressure, heart rate, temperature, and oxygen saturation were determined before the intratumoral injection, and blood pressure and oxygen saturation were monitored continuously throughout the procedure. When indicated, patients were maintained under moderate conscious sedation (26 of 30 procedures, 87%).

**Figure 2:**
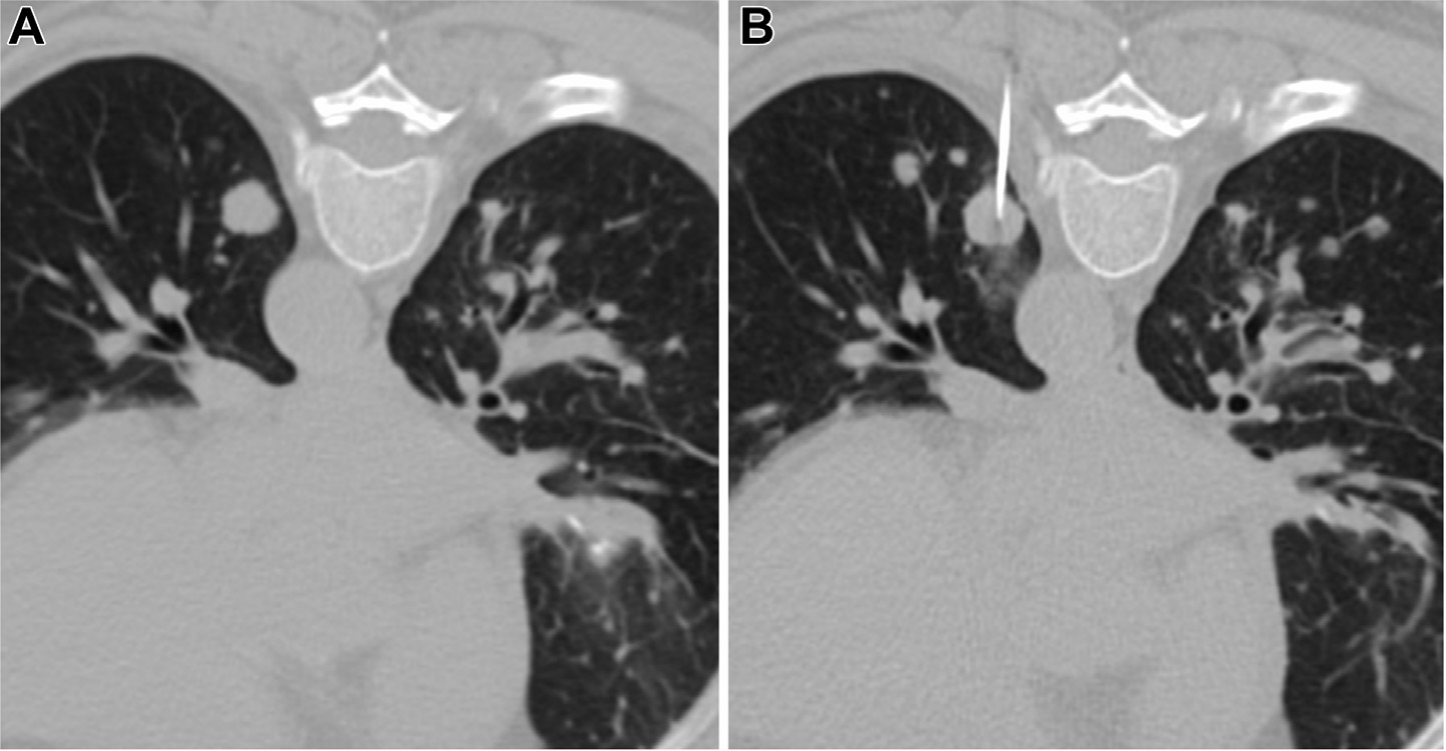
A 59-year-old male with a history of metastatic lung adenocarcinoma. *(A)* Preprocedural diagnostic CT shows innumerable diffuse bilateral pulmonary nodules. *(B)* The largest nodule (<3 cm) in the left lower lobe was targeted at its center in which seven core biopsies were obtained followed by injection of CCL21 dendritic cells. CT, computed tomography.

**Figure 3:**
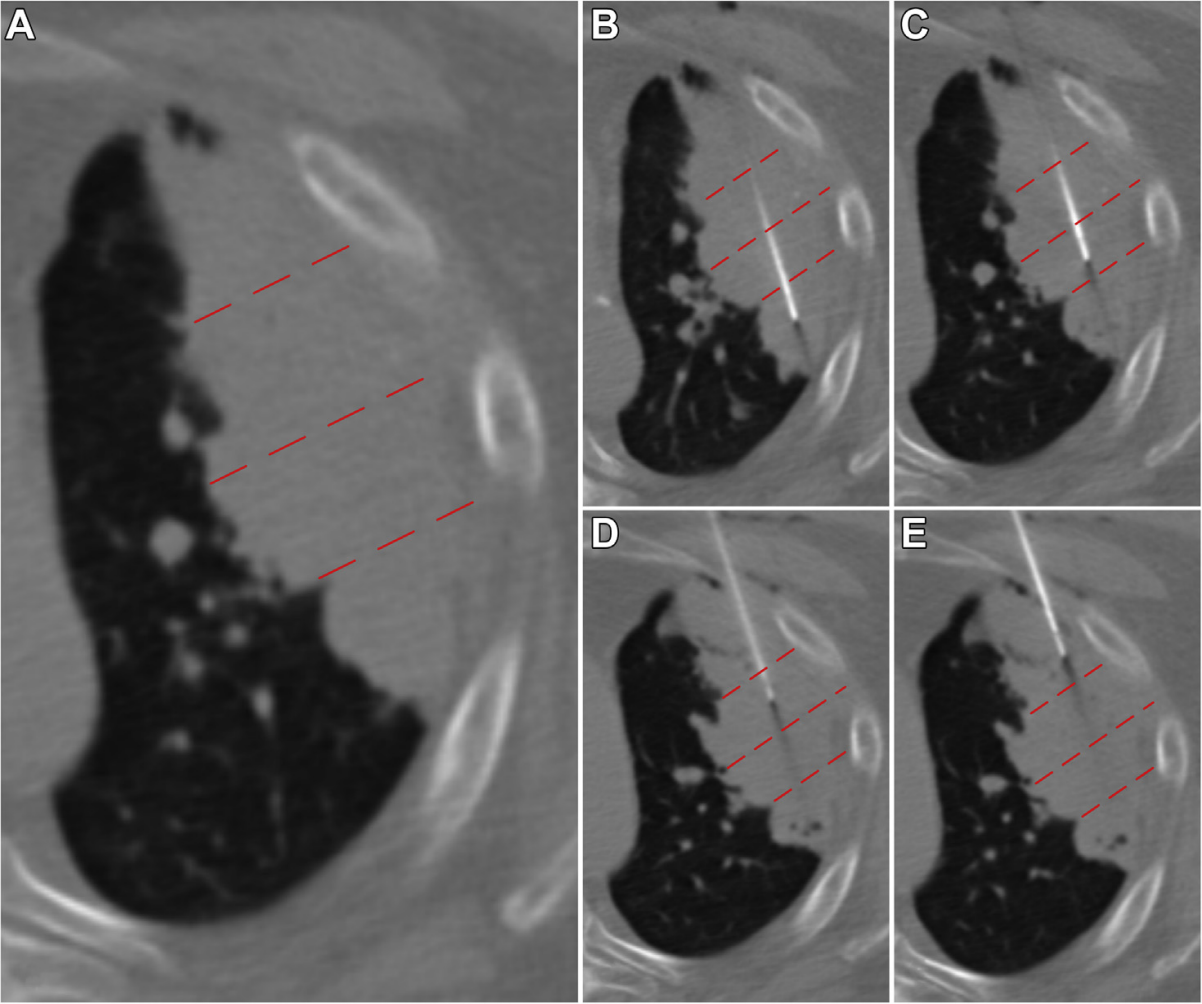
A 70-year-old male with a history of multifocal recurrent lung adenocarcinoma. *(A)* Preprocedural diagnostic axial CT illustrates large (>3 cm) left upper lobe adenocarcinoma, divided into four sections (red dotted lines). *(B–E)* Repetitive biopsy and injection of CCL21 dendritic cells at each of the four sections was performed. CT, computed tomography.

After localization, an Argon 19-gauge coaxial introducer needle (Argon Medical Devices, Frisco, TX) was placed into the target lesion. Patients with maximum lesion diameter between 1 and 3 cm were biopsied and injected at a single site (n = 4) (Fig. 2*A* and *B*), whereas patients with maximum lesion diameter greater than 3 cm were partitioned into four sections (n = 11), with deep to superficial biopsies and injections at each of the four sections (Fig. 3*A–E*). The tip was situated deep to allow for biopsy with a 20-gauge cutting needle. After at least three adequate core samples were obtained for each site, a 20- or 22-gauge Chiba needle (Cook, Bloomington, IN) was placed through the outer cannula, centering the tip within the area of interest to perform the intratumoral injection. In large heterogeneous tumors, the injection was directed to the most solid portion of the tumor. Once needle tip localization within the lesion was confirmed, gentle aspiration was performed to exclude intravenous placement. A total of 1 mL of Ad-CCL21-DC preparation was injected, either at a single site or divided evenly into four sections, as appropriate, followed by 1 mL of normal saline flush.

In cases of transpleural and intrapulmonary needle traversal (n = 9), the distance from the lesion to the visceral pleural surface was measured, and 3 to 10 mL of autologous blood was evenly injected along the entire parenchymal tract on exit, serving as a blood patch.

### Safety Profile

The primary end point for this study was defined as: (1) a safety and feasibility profile similar to that of single-needle biopsy, and (2) an absence of serious adverse events, defined as a grade greater than or equal to three according to the National Cancer Institute Common Terminology Criteria for Adverse Events version 5.0.^20^ After the Ad-CCL21-DC injections, a limited CT was performed after needle removal to evaluate for postprocedural complications. The patients were then observed for up to 4 hours in the recovery unit. Vital signs were monitored every 15 minutes and serial radiographs were obtained at 2 and 4 hours to evaluate for complications including pneumothorax. Procedural safety was evaluated by the interventional radiologist and referring oncologists with follow-up appointments up to 30 days after the first procedure.

### Statistical Analysis

Continuous data are reported as means and SDs and categorical data are reported as absolute values and percentages.

## Results

### Patient and Tumor Characteristics

A total of 15 patients with 15 NSCLC lesions underwent two cycles of technically successful transthoracic biopsies and Ad-CCL21-DC injections. The mean age was 66.5 plus or minus 9.1 years, and 6 of 15 patients (40%) were women. Histopathology was diagnostic in all cases and confirmed the diagnosis of NSCLC, most consistent with adenocarcinoma (n = 11), squamous cell carcinoma (n = 3), and neuroendocrine carcinoma (n = 1). Target lesions ranged in size from 2.4 to 99.1 cm^2^ (average 32.2 ± 34.5 cm^2^). The lesions were located in the right upper lobe (n = 5), right middle lobe (n = 1), right lower lobe (n = 2), left upper lobe (n = 6), and left lower lobe (n = 1) (Table 1).

**Table 1:** Location and Initial Size of Target Lesions

Location	Number of Lesions	Mean Lesion Size ± SD (cm^2^)^a^	Lesion Size Range (cm^2^)
Right upper lobe	5	42.7 ± 37.2	2.6–87.4
Right middle lobe	1	99.1	N/A
Right lower lobe	2	8.5 ± 8.2	2.8–14.3
Left upper lobe	6	25.3 ± 27.2	3.8–67.2
Left lower lobe	1	2.4	N/A

CT, computed tomography; N/A, not applicable.

a Lesion size was calculated as the product of nodule diameters measured on a CT scan.

For tumors between 1 and 3 cm (n = 4), five to seven adequate core biopsy samples were obtained per cycle followed by an injection distributed evenly over the entire volume (Fig. 2). For tumors greater than 3 cm (n = 11), four partitions of each lesion were biopsied at each cycle, yielding at least three adequate core biopsy samples (=12 biopsies per cycle), followed by an injection of CCL21 dendritic cells evenly divided and distributed at each section (Fig. 3). All percutaneous biopsies (N = 311) and injections (N = 96) were included in the analysis.

Tumor response as assessed by follow-up CT at 28 days revealed stability in 13 of 15 patients (87%). No procedure-related complications were identified in any of the follow-up imaging studies.

### Safety

No serious adverse events occurred related to the biopsy or Ad-CCL21-DC injection procedures in any of the 15 included patients, including hemothorax or prolonged pneumothorax. One patient withdrew after the first cycle; however, no adverse events or immediate procedure-related complications occurred for this patient within the included observation period (7 d). No patients complained of sustained pain during or after the procedure. Immediate postprocedural complications were encountered in 11 of 30 procedures (37%, all grade 1) (Table 2). The most common postprocedural complication was pneumothorax (n = 10, 33%). Two patients (7%) had small postbiopsy hemorrhage, one of whom also had a trace pneumothorax. In one patient, the pneumothorax was observed to be moderate in size during the procedure (Common Terminology Criteria for Adverse Events grade 2), which was managed with manual aspiration through the outer cannula at the conclusion of the procedure. The pneumothorax completely resolved and the outer cannula was removed. The patient remained asymptomatic, and a 2-hour follow-up chest radiograph in the recovery unit revealed no evidence of a pneumothorax. The remaining nine patients had trace pneumothoraces and were asymptomatic. All pneumothoraces either resolved or remained stable on follow-up imaging in the postprocedural observation period. A chest tube was not placed in any of the patients. All patients were in stable condition after up to 4 hours in the recovery unit and were discharged home on the same day. The two patients with small postbiopsy hemorrhage were asymptomatic and noted to have a resolution of the hemorrhage on subsequent routine CT scans in the weeks after treatment.

**Table 2:** Procedure-Related Complications

Patient (N = 15)	Injection Cycle	Complication Related to Procedure	Incidental Findings Unrelated to Procedure
1	Injection 1	Trace pneumothorax	
	Injection 2	Trace pneumothorax	
2	Injection 1	None	
	Injection 2	None	
3	Injection 1	None	
	Injection 2	Trace pneumothorax	
4	Injection 1	None	
	Injection 2	None	
5	Injection 1	None	
	Injection 2	Trace pneumothorax	
6	Injection 1	None	
	Injection 2	Trace pneumothorax	Facial swelling
7	Injection 1	Trace pneumothorax	
	Injection 2	Trace pneumothorax	
8	Injection 1	None	
	Injection 2	None	
9	Injection 1	Trace pneumothorax	
	Injection 2	Trace pneumothorax and small hemorrhage	
10	Injection 1	None	
	Injection 2	None	
11	Injection 1	None	
	Injection 2	None	
12	Injection 1	Moderate pneumothorax	
	Injection 2	Small hemorrhage	
13	Injection 1	None	
	Injection 2	None	
14	Injection 1	None	
	Injection 2	None	
15	Injection 1	None	Pleural effusion
	Injection 2	None	Pleural effusion

There were no serious (grade ≥3) procedure-related adverse events up to 30 days after the first procedure. One patient reported new unilateral facial swelling between the two consecutive procedures on the contralateral side of the targeted tumor, determined to be secondary to a combination of an indwelling catheter and progression of an invading tumor on the contralateral side, and thus, determined as unrelated to the biopsies or CCL21 dendritic cell injections. Another patient was noted to have a small pleural effusion on the ipsilateral side of the tumor on the preprocedural CT, which remained stable in size during the two consecutive procedures and on follow-up CT imaging at 28 days. This effusion was determined to be unrelated to the biopsies or CCL21 dendritic cell delivery or therapy.

## Discussion

There is a strong rationale for in situ vaccination immunotherapy strategies to induce tumor-specific immune responses that facilitate tumor antigen uptake, presentation, and T-cell activation in combination with checkpoint inhibition therapy for NSCLC.^17^ Our group previously reported on the first-in-man administration of CCL21 and the first trial of transthoracic, intratumoral in situ vaccination with Ad-CCL21-DC in the treatment of locally advanced or metastatic NSCLC.^17^ In the phase 1 trial, we reported that intratumoral injection of gene-modified dendritic cells induced a systemic tumor-specific immune response, enhanced tumor CD8^+^ T-cell infiltration (54% of all patients), and increased tumor programmed death-ligand 1 expression.^17^ Although image-guided needle placement and biopsy of lung nodules and masses is widely accepted and the risks and complications are low,^21^, ^22^, ^23^, ^24^, ^25^ the safety and complications associated with repeated transthoracic biopsies and injections of the lung are poorly understood. Here, we report that image-guided, transthoracic, intratumoral biopsy followed by immediate administration of gene-modified cell-based vaccines is safe, technically feasible, and reproducible.

This study achieved a standard of safety similar to that of single-needle biopsy, with an absence of serious (grade ≥3) adverse events. There were no new safety signals identified in our study population. Ten out of 30 patients (33%) in this study had a postprocedural pneumothorax, similar to that reported in the literature.^26^^,^^27^ Chest tube placement for evacuation of pneumothorax was not necessary for any patient. This may have been secondary to prompt diagnosis and early preventative management of pneumothorax with autologous blood patch and manual aspiration when indicated. In one patient, the moderate-sized pneumothorax was manually aspirated with the 19-gauge introducer needle at the conclusion of the procedure, with stable resolution observed before discharge from the recovery unit. Although two patients developed small postbiopsy hemorrhage, these were self-limited, and none of the patients developed large-volume hemorrhage, hemodynamic instability, or cardiovascular compromise requiring resuscitation. Ultimately, all patients were discharged home on the same day, typically after 30 minutes preprocedural preparation, 60 minutes of procedure time, and a postprocedural observation period up to 4 hours. Although there is limited data in the literature, the findings of this study are consistent with pooled meta-analyses that did not report a higher needle-related complication rate with multiple needle passes for lung biopsy using coaxial needle systems.^21^^,^^23^

Limitations in our study included small population size and a limited number of injections and biopsies (two cycles per patient, 30 cycles total). However, a large number of sites were repetitively sampled and injected, yielding more than 311 biopsies and 96 injections. Although this study presents limited data on efficacy and long-term adverse effects, our group is currently conducting a phase 1 trial of combination Ad-CCL21-DC and checkpoint inhibition in patients with stage IIIB and IV NSCLC.^28^ Another limitation is that tumors less than 1 cm were excluded as they were nonmeasurable by Response Evaluation Criteria in Solid Tumors 1.1 criteria and deemed too small to contain the entire dose, and the feasibility of repeatedly accessing the same site across separate procedures in the x, y, and z-axes was also considered in lesion selection. Future studies could be performed on smaller tumors despite the risk of leakage from tumor target given the revealed safety profile of CCL21 dendritic cell injections in our study. In addition, inaccessible tumors (e.g., bullae in needle path) were excluded from the trial, as is common practice for transthoracic procedures.^26^ In clinical practice, however, coexisting emphysema and bullae are common in patients with lung cancer.^29^ This represents a true limitation with the transthoracic approach, and continued care should be exercised when targeting these lesions, especially to avoid intractable pneumothorax or bronchopleural fistula.^30^

In conclusion, our study revealed that sequential CT-guided, transthoracic percutaneous intratumoral biopsies, and Ad-CCL21-DC vaccine injections can be administered safely and reproducibly, and with technical success. Minor complications such as pneumothorax and small hemorrhage can be expected and managed effectively at the time of the procedure or conservatively with short-term close monitoring, with subsequent outpatient follow-up. Further investigation is underway assessing the safety and efficacy of in situ vaccination with Ad-CCL21-DC in combination with anti–programmed cell death protein-1 checkpoint blockade for metastatic NSCLC (Clinicaltrials.gov: NCT03546361).^31^

## CRediT Authorship Contribution Statement

**Puja Shahrouki:** Formal analysis, Investigation, Writing - review & editing, Visualization.

**Jay M. Lee:** Conceptualization, Methodology, Investigation, Resources, Supervision.

**Jonathan Barclay:** Investigation, Writing - review & editing.

**Sarah N. Khan:** Investigation, Writing - original draft, Writing - review & editing.

**Scott Genshaft, Fereidoun Abtin, Steven M. Dubinett, Aaron Lisberg, Sherven Sharma, Edward B. Garon:** Investigation, Writing - review & editing, Resources.

**Robert Suh:** Conceptualization, Methodology, Investigation, Resources, Visualization, Supervision.
