# Supplementary material for: Technical Feasibility and Safety of Repeated Computed Tomography–Guided Transthoracic Intratumoral Injection of Gene-Modified Cellular Immunotherapy in Metastatic NSCLC
Source: JTO Clin Res Rep. 2021 Oct 14;2(11):100242. doi: 10.1016/j.jtocrr.2021.100242 (PMC8581369; doi:10.1016/j.jtocrr.2021.100242)
Supplement: Supplementary Table 1 [file mmc1.docx]

| Inclusion criteria | Exclusion criteria |
| --- | --- |
| - Adults over the age of 21 capable of giving informed consent. - Pathologically proven NSCLC with Stage IIIb, IV, or recurrent or progressive disease despite appropriate chemotherapy. - Measurable metastatic disease by RECIST Guidelines. - ECOG performance status of 0, 1, or 2. - Progressive disease despite one or more prior chemotherapy regimens as standard of care or patient’s refusal to receive standard chemotherapy. - Normal renal function (defined as BUN≤ 40 or serum creatinine ≤ 2). - Normal liver function (defined as serum total bilirubin ≤ 1.5, or serum transaminases ≤ 2.5X the upper limits of normal (ULN)). - No evidence of coagulopathy (defined as PT and/or PTT ≤ 1.5X ULN or platelets ≥ 100,000). - No evidence of leukopenia (defined as absolute neutrophil count ≥ 1,500/mm^3^). - Negative pregnancy test prior to initiation of treatment and adequate contraception throughout treatment. - All subjects must demonstrate no evidence of respiratory failure (defined as SaO_2_ >90% on room air; PCO_2_ <45mmHg; or FEV_1_ >1.0 liter). - Patients with a major endobronchial lesion in the segmental, lobar, or mainstem bronchus with complete obstruction of the airway may be eligible for bronchoscopic injection if there is no evidence of respiratory failure (defined as SaO_2_ >90% on room air; PCO_2_ <45mmHg; or FEV_1_ >1.0 liter). - Patients with an endobronchial lesion in the segmental bronchus with variable stenosis (not completely obstructed) and not amenable to standard palliative airway treatments (i.e. laser and stenting) may be eligible for bronchoscopic injection if there is no evidence of respiratory failure (defined as SaO_2_ >90% on room air; PCO_2_ <45mmHg; or FEV_1_ >1.0 liter). - Subjects with bullous disease may undergo CT-guided transthoracic injection if the targeted tumor has an intended needle path without crossing bullae. | - Radiation therapy, chemotherapy, non-cytotoxic investigational agents, or corticosteroids within the past 30 days. - Evidence of NYHA class III or greater cardiac disease, or history of myocardial infarction within the last 12 months. - Comorbid disease or a medical condition that would impair the ability of the patient to receive or comply with the study protocol. - Any use of corticosteroids within 30 days of treatment or during treatment. - Renal insufficiency (defined as BUN>40 or serum creatinine>2). - Liver insufficiency (defined as serum total bilirubin > 1.5, or serum transaminases > 2.5X the upper limits of normal (ULN)). - Coagulopathy (defined as PT and/or PTT > 1.5X ULN or platelets < 100,000). - Leukopenia (defined as absolute neutrophil count < 1,500/mm^3^). - Respiratory failure (defined as SaO_2_ <90% on room air; PCO_2_ >44mmHg; or FEV_1_ <1.0 liter) - Acute viral, bacterial, or fungal infection, which requires specific therapy. Acute therapy must have been completed within 14 days prior to study treatment. - HIV infected patients, due to concerns in the ability to stimulate an effective immune response. - Hypersensitivity to any reagents used in the study. - Pregnancy or inadequate contraception. - Lactating females. - Active CNS metastasis (progression of CNS disease during the 30 days without intervention). - Subjects with organ allografts. - Subjects exhibiting signs or symptoms of acute adenoviral infection (i.e. conjunctivitis or documented adenoviral upper respiratory infection). - Previous or concurrent evidence of autoimmune disease. - Patients with a major endobronchial lesion in the lobar or mainstem bronchus amenable to standard palliative airway treatments or with >50% stenosis (not completely obstructed airway) will be excluded from bronchoscopic injection. - Subjects with bullous disease may not undergo CT-guided transthoracic injection if the targeted tumor has an intended needle path that requires crossing the bullae. |

**Supplementary Table 1. Inclusion and exclusion criteria**
